# Relationship between health-related quality of life, and acute care re-admissions and survival in older adults with chronic illness

**DOI:** 10.1186/1477-7525-11-136

**Published:** 2013-08-06

**Authors:** Anastasia Hutchinson, Tshepo Mokuedi Rasekaba, Marnie Graco, David John Berlowitz, Graeme Hawthorne, Wen Kwang Lim

**Affiliations:** 1 Northern Clinical Research Centre, Northern Health & Department of Medicine, The University of Melbourne, Melbourne, Australia; 2 Institute for Breathing and Sleep, Austin Health, Melbourne, Australia; 3 Mental Health Evaluation Unit, Department of Psychiatry & Northern Clinical Research Centre, Department of Medicine, University of Melbourne, Melbourne, Australia; 4 Department of Medicine and Aged Care, Northern Health & Department of Medicine, The University of Melbourne, Melbourne, Australia; 5 Northern Clinical Research Centre, Northern Health, 185 Cooper Street, Epping 3076, Victoria, Australia

**Keywords:** Health-related quality of life, Chronic disease, Case management, Mortality, Health care utilisation

## Abstract

**Background:**

Australia’s ageing population means that there is increasing emphasis on developing innovative models of health care delivery for older adults. The assessment of the most appropriate mix of services and measurement of their impact on patient outcomes is challenging. The aim of this evaluation was to describe the health related quality of life (HRQoL) of older adults with complex needs and to explore the relationship between HRQoL, readmission to acute care and survival.

**Methods:**

The study was conducted in metropolitan Melbourne, Australia; participants were recruited from a cohort of older adults enrolled in a multidisciplinary case management service. HRQoL was measured at enrolment into the case-management service using The Assessment of Quality of Life (AQoL) instrument. In 2007–2009, participating service clinicians approached their patients and asked for consent to study participation. Administrative databases were used to obtain data on comorbidities (Charlson Comorbidity Index) at enrolment, and follow-up data on acute care readmissions over 12 months and five year mortality. HRQoL was compared to aged-matched norms using Welch’s approximate t-tests. Univariate and multivariate logistic regression models were used to explore which patient factors were predictive of readmissions and mortality.

**Results:**

There were 210 study participants, mean age 78 years, 67% were female. Participants reported significantly worse HRQoL than age-matched population norms with a mean AQOL of 0.30 (SD 0.27). Seventy-eight (38%) participants were readmitted over 12-months and 5-year mortality was 65 (31%). Multivariate regression found that an AQOL utility score <0.37 (OR 1.95, 95%CI, 1.03 – 3.70), and a Charlson Comorbidity Index ≥6 (OR 4.89, 95%CI 2.37 – 10.09) were predictive of readmission. Multivariate analysis demonstrated that age ≥80 years (OR 7.15, 95%CI, 1.83 – 28.02), and Charlson Comorbidity Index ≥6 (OR 6.00, 95%CI, 2.82 – 12.79) were predictive of death.

**Conclusion:**

This study confirms that the AQoL instrument is a robust measure of HRQoL in older community-dwelling adults with chronic illness. Lower self-reported HRQoL was associated with an increased risk of readmission independently of comorbidity and kind of service provided, but was not an independent predictor of five-year mortality.

## Background

Australia’s ageing population means that there is increasing demand on acute health care services and greater emphasis is being placed on innovative community-based models of health care delivery for older adults. The assessment of the most appropriate mix of services for an individual and measurement of whether such services have had a positive impact on patient outcomes is a challenging issue; especially since over 80% of those aged over 65 years have three or more chronic health conditions (i.e. a condition lasting more than 6-months) [1]. Under these circumstances the use of disease-specific outcome measures to assess the impact of health interventions on health-related quality of life (HRQoL) may be inappropriate as comorbidities may be ignored and/confound results. Additionally, issues of validity confound the use of disease-specific measures as these instruments emphasise the impact of disease symptoms rather than HRQoL as a holistic construct. When models of care are predominantly focused on coordination and access to social care services rather than disease-specific symptom management, there is a need to use instruments that measure HRQoL as a holistic construct.

In contrast to disease-specific measures, generic HRQoL instruments aim to measure the impact of an individual’s health on important aspects of their lives (including psychological well-being, independence, social functioning) and may be more appropriate measures of overall improvements. The Assessment of Quality of Life (AQoL) [2] is one generic instrument that has been developed for and validated in the Australian population [3,4] and has been demonstrated to be a sensitive measure of HRQoL in community dwelling older adults [5].

The Northern Alliance Hospital Admission Program (NA-HARP) complex needs service provides care to a socio-economically disadvantaged population living in the northern metropolitan region of Melbourne, Australia [6]. It includes a high proportion (approximately 60%) of individuals whose first language is other than English. The purpose of the NA-HARP program is to decrease the need for acute care admission by optimising medical and social care within a community setting. At enrolment into the service a comprehensive assessment is performed by a member of the multidisciplinary team to ensure that clients are offered a ‘package of care’ that is most appropriate to their needs. This study was undertaken to evaluate whether including a measure of HRQoL at the initial assessment would provide clinicians’ with information about their future prognosis and demand for acute health care services that would be useful when planning care.

The aim of this evaluation was to describe the HRQoL of older adults with complex needs and to explore the relationship between HRQoL, readmission to acute care and survival. We hypothesised that individuals with lower self-reported HRQoL would have greater acute health care utilisation and higher mortality than those within the normal age–adjusted range.

## Methods

### Setting

The Northern Alliance Hospital Admission Program (NA-HARP) complex needs service offers multidisciplinary case management and care coordination for older clients (typically ≥60 years) with complex health needs that put them at high risk of requiring acute care admission. The service included two models of care: (a) a rapid assessment and care coordination service (RAC) for patients recently discharged from acute care and (b) a community case-management (CCM) and support service for high-risk older adults living independently in the community. The RAC provided access to geriatrician review for unstable medical problems and short-term case-management. The CMM service provides long-term case-management within a community care setting with medical management provided by the clients’ primary care physicians.

#### Study design

A prospective, longitudinal cohort design was used to evaluate the impact of HRQoL on 12 month readmission rates and five year mortality [6]. Baseline data was collected from September 2007 to 2009 and follow-up data was obtained until December 2012. This project was approved by the Northern Health institutional human research ethics committee, the requirement for written informed consent was waivered; but patients included in this study gave verbal consent to study participation.

#### Participants

Participants were patients enrolled in the NA-HARP complex needs service that had given verbal consent to study participation and completed the AQoL at program enrolment.

### Data collection

From 2007 to 2009, NA-HARP clinicians approached their patients and obtained verbal consent for study participation. Surveys were either distributed by mail or given to participants following their first assessment visit with the service to facilitate study participation and self-completion of the AQoL. Surveys were returned to the service by reply paid post. Although the AQoL has been translated into several languages, professional interpreters were made available to assist participants who spoke a language other than English and those with limited literacy.

#### Outcomes

The AQoL is a validated, multi-attribute utility instrument designed to assess HRQoL that is sensitive to a range of patient conditions and care models [2]. It measures five dimensions: “Illness”, “independent living”, “social relationships”, “physical senses”, and “psychological wellbeing”. These scales are scored as proportions on a 0.00-1.00 scale. Scores from the last four dimensions are combined using a multiplicative model weighted with community values to compute the utility index, which is suitable for use in cost-utility analysis [2]. The utility scores range from −0.04 (HRQoL worse than death), 0.00 (representing death equivalent states), to 1.00 (full HRQoL). The AQOL is designed to be self-administered taking an average of five to seven minutes to complete. Population norms are available, which allow the results to be interpreted relative to the age-matched average Australian population [3,4]. The published minimum important difference (MID) is 0.06 utilities [3].

Administrative data was used to classify patients’ primary reasons for enrolment into the NA-HARP service according to ICD-10 codes [7,8]. The Charlson Comorbidity score (Charlson) at baseline was calculated based on patients’ primary and secondary ICD-10 diagnoses codes from acute care admissions prior to the patient’s enrolment in the NA-HARP complex needs service. Charlson weights were allocated to ICD-10 scores using the algorithm developed by Quan e al. [9,10].

At the end of the follow-up period, the number of readmissions to acute care in the 12-months following enrolment and five year mortality data were obtained from the regional health service’s administrative dataset and verified by audit of individual patient medical records. Data was obtained on both the number and time (measured in years) to these outcomes.

#### Data analysis

Administrative and AQoL data were retrieved for patients enrolled in the service between September 2007 and September 2009. Continuous data were summarised as means and standard deviations (SDs); categorical data as frequencies and percentage, differences in proportions were analysed with Chi-square (χ^2^) tests, differences in continuous outcomes using T-tests and ANOVA.

Examination of AQoL utilities revealed that the data were non-normally distributed. Prior to statistical analysis, AQoL scores were transformed to remove data skew, although untransformed means and SDs are reported in the interests of readability. Missing AQoL item data were imputed using horizontal mean imputation, restricted to <30% of items. Differences in baseline AQoL utilities were analysed with analysis of variance (ANOVA). Mean AQoL scores were compared with published population data across age deciles using Welch’s approximate t-tests to control for differences in data distributions [11]. Differences between the aged care services (RAC, CCM) in baseline AQoL scores and 12-month readmission rates were compared using independent t-tests. Statistical significance was set at p < 0.05. Univariate logistic regression was used to assess which factors were predictive of 12-month re-admissions and five year mortality [12]. AQoL scores were dichotomized at two standard deviations below the population norm (ie: (<0.37/≥0.37) [4]. and Charlson scores were dichotomized at the cut-point for the highest quartile (≤5/6-15) [10]. Multivariate logistic regression, using a forward stepwise model, was used to assess whether lower AQoL scores at enrolment were predictive of patients who required readmission within 12-months of enrolment and five year mortality, after adjusting for factors predictive in the univariate analysis. In the secondary analysis the AQoL was replaced iteratively with each of the AQoL dimensions. In the absence of any known cut points, the dimensions were entered as continuous variables into the model.

#### Sample size

The study sample size was calculated according to the methodology described by Davison et al. [13]. Previous studies of older adults have reported mean AQoL scores of 0.30-0.45 (5, 8–10) and 0.02-0.20 for hospitalized older adults [14,15]. Taking the lower estimate for community-residing older adults (0.30) and the upper estimate for hospitalized older adults (0.20), it was apparent that a greater change in AQoL scores would be needed to predict hospitalization than the published minimum important difference of 0.06 [3]. The difference between community-residing and hospitalized older adults was therefore accepted as the critical change score. Using Kazis’ effect size [16], estimated at 0.42 based on the literature above, and Davison et al’s [12] formula for sample sizes, the calculated sample size was 122 cases. This was then adjusted for the expected intracluster correlation coefficient (ICC) given that participants were recruited through two services (i.e. participants were clustered samples). To estimate the ICC we used the mid-point of SF-36 scale ICCs (0.08) [17] and calculated the design effect after Hsieh [18] to be 1.16; this gave a calculated sample size of 142 participants.

The data were analysed using PSAW Statistics 18.0 [19] and STATA version 11, Statacorp Texas USA [20].

## Results

During the study recruitment period, 2609 individuals were enrolled in the NA-HARP Complex needs program of whom 210 (8%) were enrolled in the study. The mean follow-up time for study participants was 2.71 years (range 0.01 to 5.4 years). Participants were mostly female with an average age of 78 years, 52% were born in a country other than Australia, 26% spoke a language other than English, and 54% lived with their families (Table 1). Comparison with non-participants showed that there were no statistically significant differences in age (mean 78 (SD 8.6 years) vs. 78 (SD 7.8 years); t = 0.80, df = 2607, p = 0.43). Study participants were however, more likely to be: female (67% vs. 58%, χ^2^ = 5.84, df = 1, p = 0.02), living alone (χ^2^ = 4.59, df = 1, p = 0.03); born overseas (χ^2^ = 185.39, df = 1, p < 0.01); and were less likely to need the services of an interpreter (χ^2^ = 11.71, df = 1, p <0.01).

**Table 1:** Participant characteristics

***N. participants***		***210***
Gender	Female	67%
Age	Mean (SD) years	78 (7.8)
Country of birth	Australia	52%
Primary language spoken	English	74%
Interpreter used for interview		17%
Accommodation	Lived alone	39%
	Lived with family	54%
	Lived with others	7%
Caregiver status	Caregiver	16%
Primary health condition (a)	Cancer	11%
	Cardiac	23%
	Mental illness	23%
	Muscular/Pain	26%
	Respiratory	9%
	Other	10%
Charlson Comorbidity Score	0/1	43%
	2/3	21%
	4/5	13%
	≥6	22%

a = Referral reason coded by ICD-10.

Sixty-three (30%) participants were enrolled in the CCM and 147 (70%) in the RAC service. The major reasons for enrolment in the NA-HARP complex needs program were: functional impairment and musculoskeletal conditions (36%), cognitive impairment or neurological conditions (17%), chronic medical conditions (39%) and other issues that required case management support (7%), (Table 1). The Charlson showed that 56% of participants had significant co-morbidities, and that this varied significantly by service: 27% of those in RAC versus 11% of those in CCM obtained Charlson scores ≥6 (χ^2^ = 9.06, df = 3, p = 0.03). There were no other statistically significant differences by Charlson. There were 4 (2%) patients less than 60 years included in the study, these patients had been referred to NA-HARP for case-management of complex or severe disease (Parkinson’s Disease (1), severe functional impairment secondary to obesity (2), severe chronic obstructive pulmonary disease (1)); the average Charlson score in this group was 5 (range 3–11).

The mean HRQoL for all participants was AQoL 0.30 (SD 0.27). When compared with age-matched population norms (4), participants reported significantly worse HRQoL. For those aged 60–69 years this decrement was 0.66 utilities, for those aged 70–79 years it was 0.44 utilities and for those aged 80+ years it was 0.40 utilities (Table 2). These differences exceeded the published minimum important difference (MID 0.06) for the AQoL across all three age groups included in this study.

**Table 2:** **Participants**’ **HRQoL compared with age**-**adjusted population values**

***Age group***	***Participants***			***Population (a)***			***Statistics (b)***
	***N***	***Mean***	***SD***	***N***	***Mean***	***SD***	
60-69 years (c)	27	0.26	0.28	1245	0.80	0.22	t = 13.28, df = 326,***
70-79 years	88	0.33	0.29	912	0.76	0.23	t = 13.09, df = 349, ***
80+ years (d)	93	0.30	0.26	357	0.70	0.26	t = 8.98, df = 187,***

Notes:

a = Source: Hawthorne et al. (In press).

b = Welch’s approximate t, p-values: * <0.05, ** <0.01, *** < 0.001.

c = Includes 4 cases <60 years.

d = the population sample was restricted to those aged 80–85 years.

Table 3 shows the HRQoL of participants by age group, medical condition, Charlson score and service type. There were statistically significant differences across age groups in both the physical senses and psychological wellbeing dimensions of the AQoL. For the physical senses dimension those aged ≥80 years obtained scores indicating loss of physical senses (seeing, hearing and communication ability); in contrast for the younger age group aged 60–69 years the psychological well-being dimension indicated poorer mental health (anxiety, sleep quality and pain). There were no statistically significant differences in overall AQoL utilities by age group, although the difference between those aged 60–69 years and those aged 70–79 years exceeded the published MID of 0.06 [3].

**Table 3:** **AQoL dimension scores and utilities at study enrolment**, **by age group**, **primary health conditions and service type**

		***N***	***Mean AQoL dimension scores***					***AQoL utility *( *c *)**
		**( *a *)**	**( *b *)( *c *)**					
			***Ill***	***IL***	***SR***	***PS***	***PW***	
Age group	60-69 years	27	0.23 (0.26)	0.48 (0.33)	0.64 (0.31)	0.88 (0.12)	0.66 (0.28)	0.25 (0.28)
	70-79 years	89	0.23 (0.25)	0.52 (0.34)	0.72 (0.27)	0.88 (0.13)	0.77 (0.19)	0.32 (0.28)
	≥80 years	94	0.25 (0.25)	0.48 (0.32)	0.72 (0.30)	0.80 (0.17)	0.78 (0.20)	0.30 (0.26)
**Statistics (d)**	**ANOVA**		**F = 0.07**	**F = 0.40**	**F = 0.88**	F = 7.38***	F = 3.93*	**F = 1.14**
						**P ≤ 0.001**	**P ≤ 0.05**	
Primary health condition (e)	Cancer	23	0.35 (0.26)	0.62 (0.28)	0.83 (0.19)	0.86 (0.13)	0.82 (0.11)	0.42 (0.24)
	Cardiac	47	0.20 (0.22)	0.40 (0.32)	0.69 (0.26)	0.78 (0.17)	0.76 (0.22)	0.24 (0.26)
	Mental illness	46	0.18 (0.18)	0.50 (0.35)	0.69 (0.32)	0.81 (0.20)	0.73 (0.24)	0.30 (0.29)
	Muscular/Pain	51	0.22 (0.26)	0.43 (0.30)	0.68 (0.31)	0.87 (0.14)	0.78 (0.21)	0.27 (0.25)
	Respiratory	18	0.25 (0.31)	0.63 (0.29)	0.81 (0 l.27)	0.87 (0.08)	0.77 (0.15)	0.39 (0.27)
	Other	21	0.32 (0.25)	0.56 (0.34)	0.61 (0.32)	0.88 (0.13)	0.74 (0.20)	0.32 (0.29)
**Statistics (d)**	**ANOVA**		F = 2.30*	F = 2.87*	**F = 1.95**	**F = 2.18**	**F = 0.54**	F = 2.32*
Charlson	0/1	90	0.25 (0.23)	0.49 (0.32)	0.75 (0.26)	0.83 (0.16)	0.79 (0.17)	0.32 (0.26)
	2/3	45	0.27 (0.28)	0.59 (0.031)	0.72 (0.30)	0.86 (0.16)	0.76 (0.24)	0.36 (0.28)
	4/5	26	0.22 (0.22)	0.38 (0.36)	0.62 (0.32)	0.82 (0.19)	0.75 (0.19)	0.24 (0.28)
	≥6	47	0.16 (0.22)	0.45 (0.30)	0.64 (0.31)	0.84 (0.15)	0.73 (0.25)	0.24 (0.26)
**Statistics (d)**	**ANOVA**		F = 2.87*	F = 2.69*	**F = 2.35**	**F = 0.52**	**F = 0.60**	F = 2.78*
Service (f)	CCM	62	0.25 (0.24)	0.62 (0.29)	0.81 (0.23)	0.85 (0.15)	0.80 (0.18)	0.42 (0.26)
	RAC	146	0.23 (0.25)	0.43 (0.32)	0.66 (0.30)	0.83 (0.16)	0.75 (0.22)	0.25 (0.26)
**Statistics (d)**	**Independent *t***-**test**		**t = −0.80**	t = 4.06***	t = 3.61***	**t = −0.65**	**t = 1.29**	t = −4.52***

Notes:

a = Missing data is the difference between 147-(Sum of Ns. for each analysis).

b = *Ill* Illness, *IL* Independent living, *SR* Social relationships, *PS* Physical senses, *PW* Psychological wellbeing.

c = All scores are means and SDs.

d = Transformed data, p-values: * ≤ 0.05; ** ≤ 0.01; *** ≤ 0.001.

e = Based on ICD-10 classifications.

f = *CCM* Community case management, *RAC* Rapid assessment and care coordination.

Participants enrolled in the RAC had statistically significantly lower AQoL utilities compared to the CMM service, as did those whose primary health problem was cardiac or muscular/pain (Table 3). Multivariate linear regression found that this difference by service (RAC/CMM) remained significant after adjusting for differences in age, gender and co-morbidities (Charlson) between the two groups (standardised β_Adj_ = 0.28, p < 0.001). There were no statistically significant differences in AQoL utilities by age, gender or co-morbidities (Charlson). When AQoL dimensions were examined, there were statistically significant differences by service type for the independent living and social relationships dimensions with those enrolled in the RAC obtained scores indicating poorer HRQoL than those in CCM (Table 3).

Seventy-eight (38%) participants were readmitted during the 12-months following enrolment. The number of readmissions ranged from one through to 15. Of readmitted cases, 63 (82%) were enrolled in the RAC; those in this service had almost three-times the odds of readmission of those in the CCM service (OR = 2.72 (95%CI: 1.38-5.37)). Additionally, those with Charlson scores 6–15 were significantly more likely to be readmitted, with over five-times the odds of readmission compared with those with Charlson scores ≤5 (OR = 5.33 (95%CI: 2.64-10.76)). There were no statistically significant differences in readmission status by age, gender or primary health condition.

A logistic regression model was constructed to predict hospital re-admission (No/Yes). The three statistically significant variables discussed above were included in the initial model; service type (CMM/RAC), Charlson score (≤5/6-15) and AQoL (<0.37/≥0.37). Two significant predictors of readmission in the 12-months following enrolment were observed. Those with Charlson scores ≥6 had odds of readmission that were five-times that of those with scores ≤5, and those with AQoL utilities <0.37 had two-times greater odds of readmission compared with those with higher AQoL scores. In the multivariate model service type was not a significant predictor of readmission (Table 4). The AQoL was replaced with each of the AQoL dimensions, iteratively. The only AQoL dimension which statistically predicted readmission was psychological wellbeing (β_adj_ = −2.02, p = 0.01), indicating that those with higher scores were less likely to be admitted.

**Table 4:** **Multivariate logistic regression models**: **predicting hospital readmission and death**

***Predictor***	***Base***	***Contrast***	***OR***	***95%CI***
**Predicting hospital re**-**admission (12-months post-enrolment)**				
AQoL utility scores	≥0.37	<0.37	1.95	1.03 – 3.70
Charlson	≤5	≥6	4.89	2.37 – 10.09
Service	CCM	RAC	1.78	0.85 – 3.73
Logistic regression, correct classification: 71.4%; Hosmer and Lemeshow *χ*^2^ = 0.17, p = 0.99.				
**Predicting death (5-years post-enrolment)**				
Age	60-69 years	70-79 years	3.88	0.99 – 15.22
		≥80 years	7.15	1.83 – 28.02
AQoL utility scores	≥0.37	<0.37	1.61	0.79 – 3.25
Charlson	≤5	≥6	6.00	2.82 – 12.79

Logistic regression, correct classification: 75.0%; Hosmer and Lemeshow *χ*^2^ = 2.23, p = 0.90.

By the end of follow-up 65 (31%) of study participants had died, the mean age of survivors was 77.4 years (range 50–93) and of those who died 80.0 years (range 62–100). The mean follow-up time in survivors was 2.85 years (range 0.01 to 5.41) versus mean time to death 2.3 years (range 0.01-5.2), p = 0.025. In univariate analysis, five-year mortality was predicted by age group: when compared with those aged 60–69 years there was no statistically significant increased odds of dying for those aged 70–79 years (OR: 3.30; 95%CI: 0.91 – 11.93), whereas those aged ≥80 years had an odds of death five-times greater than those aged 60–69 years (OR: 4.97; 95%CI: 1.39 – 17.69). Death was also predicted by Charlson score: those with scores ≥6 had over five-times greater odds of dying compared with those with Charlson scores <6 (OR: 5.68; 95%CI: 2.84 – 11.39); and by the dichotomized AQoL: those with scores <0.37 had an odds of dying two-times greater than those with higher scores (OR: 1.93; 95%CI: 1.01 – 3.68). There were no statistically significant differences by gender, primary health condition or type of service.

A logistic regression model was constructed to predict death, those aged ≥80 years had three-times the likelihood of dying compared with those aged 60–69 years and those with a Charlson score ≥6 had six-times the likelihood of dying when compared with those with Charlson scores ≤5. AQOL scores <0.37/≥0.37 were not significant in the multivariate model (Table 4).

## Discussion

The HRQoL, as assessed by the AQoL, of patients in enrolled in the NA-HARP aged care service was significantly lower than age adjusted general population norms [4] and was within the range of scores reported in the literature for older adults with acute or chronic health conditions. Lower AQoL scores were predictive of acute care readmission over the following 12 months.

Although only a small proportion of the older adults using the NA-HARP aged care services were included in this study, their HRQoL was similar to those reported in other studies of community-resident older adults with chronic illness [21-24]. The mean AQoL utility score for the whole sample was 0.30 (SD = 0.27), in contrast to the norm for general population aged 70–79 years which is 0.76 (SD = 0.23) [4]. Previous studies in older adults with chronic health conditions have found that HRQoL is typically lower than this population norm; Harris et al. [21] reported a mean AQoL utility of 0.30 (95%CI: 0.28 to 0.32) and Osborne et al. a mean utility score of 0.33 (95%CI: 0.32- 0.35) [5]. The findings from this study are consistent with these values, suggesting that older adults with chronic health conditions experience a HRQoL that is approximately half that of older adults in general.

The study participants who were enrolled in the long-term CCM program experienced better HRQoL than those enrolled in the RAC service probably reflecting differences in the acuity of illness between the two services. AQoL utility scores reported by the long-term community service (CCM) (mean 0.42) were comparable to those reported by Holland [25] and Foley [22] in studies of community-dwelling older adults (mean 0.40-0.45). In contrast the mean AQoL utility score in the post discharge arm (RAC) was lower than that reported by Lim and colleagues [23] when evaluating a post discharge case-management service, but higher than that reported in a study of frail older adults being transferred to long-term residential care (0.02–0.05) [15]. These findings confirm that the AQoL is sensitive to differences in HRQoL in older adults [26], across the spectrum from healthy older adults, community-dwelling older adults with chronic conditions [5], those recovering from acute illness [14,23], to those requiring long-term residential care [27].

Our study findings suggest that differences in overall HRQoL may be partially explained by differences in their functional capacity and psychological well-being. In this study there was a significant difference in the physical senses dimension across aged groups; probably reflecting that the impact of sensory deficits on functional capacity is greatest in the over 80 year old age group [28]. This association is reflected in studies in the rehabilitation literature that have found direct effects of disability limitations and physical self-worth on HRQoL [29]. A number of studies have documented that functional independence and the capacity to perform activities of daily living are considered highly important in determining older adults’ estimation of their quality of life [29-33]. The key losses among younger study participants were in their psychological wellbeing. It is possible that younger participants were struggling more with major life changes, (such as the development of significant health problems or loss of employment) whereas older participants may have reached acceptance of both these life stages. As individuals age, their personal priorities change and the factors that influence their psychological well-being evolve with their changed circumstances. Despite this AQoL scores did not statistically vary by Charlson comorbidity scores. A possible explanation lies in the disability paradox, which is where people with demonstrable poor health adjust their internal calibrations to report a good HRQoL [34].

A key study finding was in relation to base-line AQoL scores predicting one-year hospital readmission. This finding is consistent with Bilotta and colleagues’ study which found older adults’ assessment of overall HRQoL was independently predictive of emergency department readmissions [35]. To confirm the robustness of this association and to demonstrate that this finding is generalizable to a wider spectrum of patients admitted to acute care, this finding needs to be validated in a larger prospective cohort study of patients being discharged from acute care.

Our study found that there were significant differences between survivors and non-survivors in their baseline assessment on the independent living dimension of the AQoL instrument, but no significant differences in the other dimensions. These findings suggest that the independent living dimension of the AQoL may provide a measure of frailty which is predictive of poor prognosis [36] and poor overall HRQoL [37]. Our study found that HRQoL (measured by the AQoL) was not an independent predictor of five year mortality after adjusting for age and co-morbidities. This is in contrast to an Italian study that reported HRQoL (measured by the Older People’s Quality of Life instrument) was predictive of one year mortality after adjusting for age, frailty and co-morbidities [35]. One explanation for these seemingly contradictory findings is the difference in follow-up time between the two studies. It is likely that co-morbidities will be a stronger predictor of prognosis than HRQoL in the medium term, and that individuals assessment of their HRQoL will change as their health status worsens. Larger sample sizes than available in our study would therefore be needed to measure an independent association between HRQoL and five-year mortality [38].

The study limitations included the small number of participants as a proportion of all NA-HARP participants and differences between groups in measuring the AQoL. Readmission and mortality data were obtained from the regional health service’s administrative dataset and medical record review. It is therefore possible that this is an underestimation of these outcomes as patients who are lost to follow-up may have died. This will have decreased the statistical power of the study to detect an association between AQoL scores and mortality. As participants may have been admitted to health care providers whose data is not captured in our regional health service dataset it is also possible that this has introduced bias into the study. It is possible that individuals with lower HRQoL were also less likely to access over health care providers thereby overestimating the association between low HRQoL and 12 month readmission rates. Study participants experienced a relatively small range of primary medical conditions, thereby limiting the generalizability of the study.

## Conclusions

This study confirms that the AQoL instrument is a robust measure of HRQoL in older community-dwelling adults with chronic illness. Lower self-reported HRQoL in older adults is associated with an increased risk of hospital re-admission, but was not an independent predictor of five-year mortality in this study. Further studies are needed to validate the association between low AQoL scores and acute care readmissions.

## Abbreviations

HRQoL: Health-related quality of life; AQoL: The assessment of quality of life instrument; NA-HARP: The northern alliance hospital admission program; RAC: Rapid assessment and care coordination service; CCM: Community case-management; ICC: Intracluster correlation coefficient; MID: Minimum important difference; SD: Standard deviation; OR: Odds Ratio; 95%CI: 95% Confidence Interval.

## Competing interests

The authors declare that they have no competing interests.

## Authors' contributions

AH was involved in study implementation and data management, data analysis and manuscript preparation, TMR was involved in data management and analysis, DJB, MG & WKL contributed to study design and manuscript prepartion. GH contributed to study design, data analysis and manuscript preparation. All authors read and approved the final manuscript.
